# Endoscopic ultrasound-guided radiofrequency ablation of a mediastinal lymph node metastasis from clear cell renal cell carcinoma

**DOI:** 10.1055/a-2873-7665

**Published:** 2026-07-29

**Authors:** Carlo Covello, Ina Isufi, Joao P. Pinho, Francesco Crafa, Alberto Larghi

**Affiliations:** 1 Università Cattolica del Sacro Cuore 18654 Fondazione Policlinico Universitario A. Gemelli IRCCSRomeLazioItaly; 2 General Surgery 389794 Hospital de BragaBragaBragaPortugal; 3 Oncological, General and Robotic Surgery Unit 90384 S. Giuseppe Moscati HospitalAvellinoCampaniaItaly

Endoscopic ultrasound-guided radiofrequency ablation (EUS-RFA) is an emerging minimally invasive technique for locoregional tumor control, 1​ ​2​ mainly utilized to treat different types of pancreatic lesions. Its application has also been described for extrapancreatic lesions, including lymph node metastases, although clinical experience remains limited. 3​

Clear cell renal cell carcinoma (ccRCC) is characterized by a propensity for late metastatic spread. Mediastinal lymph node involvement is uncommon, but may represent a therapeutic challenge due to anatomical constraints and limited surgical accessibility. 4​

**Fig. 1:**
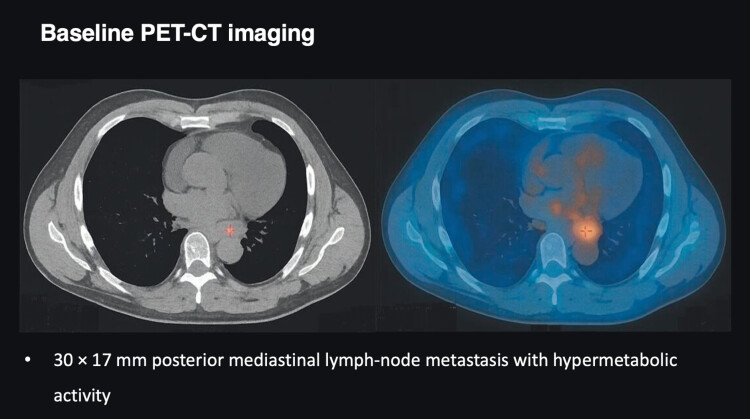
A representative image showing baseline PET-CT imaging of the posterior mediastinal lymph node metastasis.

We report the case of a 58-year-old man with a history of ccRCC who presented with a posterior mediastinal lymph node metastasis detected on positron emission tomography–computed tomography (PET-CT). Baseline imaging demonstrated a 30×17 mm lymph node with hypermetabolic activity ( Fig. 1 ). Attempted thoracoscopic resection was aborted because of anatomical inaccessibility. Therefore, patient was referred to us for EUS-RFA treatment ( Video 1 ).

**Video 1** Endoscopic ultrasound-guided radiofrequency ablation of a posterior mediastinal lymph-node metastasis from clear cell renal cell carcinoma, showing needle placement and radiofrequency.


The procedure was performed using a therapeutic linear EUS echoendoscope through the esophagus. The 19-gauge radiofrequency ablation needle was advanced into the central part of the lesion under real-time ultrasound guidance. Radiofrequency energy was delivered at 30 W, with five ablation cycles initiated in the central portion of the lymph node. During radiofrequency delivery, intralesional bubble formation was observed, consistent with tissue vaporization and effective ablation. In the final phase of the procedure, radiofrequency was used to complete ablation of the peripheral portions of the lymph node. The procedure was technically successful, and no intra- or post-procedural adverse events occurred. Potential adverse events of mediastinal EUS-RFA include bleeding, thermal injury to adjacent structures, infection, and esophageal wall perforation; these risks were minimized by Doppler assessment and controlled, stepwise RFA energy delivery.

**Fig. 2:**
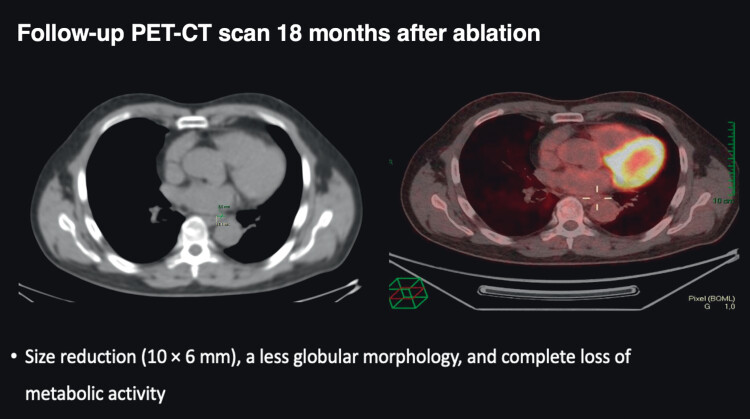
A representative image showing PET-CT imaging 18 months after ablation of the same lymph node lesion.

Follow-up PET-CT performed 18 months after the procedure demonstrated a marked reduction in the lymph node size, a less globular morphology, and complete loss of metabolic activity ( Fig. 2 ). These findings confirmed durable local tumor control.

This case illustrates the feasibility and the efficacy of EUS-RFA for a mediastinal lymph node metastasis from ccRCC in a patient unsuitable for surgery, supporting its potential role in selected anatomically challenging metastatic lesions.

Endoscopy_UCTN_Code_TTT_1AS_2AC

### Competing interests

The authors declare that they have no competing interests related to this publication.
